# Mobile Phone Sensor Correlates of Depressive Symptom Severity in Daily-Life Behavior: An Exploratory Study

**DOI:** 10.2196/jmir.4273

**Published:** 2015-07-15

**Authors:** Sohrab Saeb, Mi Zhang, Christopher J Karr, Stephen M Schueller, Marya E Corden, Konrad P Kording, David C Mohr

**Affiliations:** ^1^ Center for Behavioral Intervention Technologies Department of Preventive Medicine Northwestern University Chicago, IL United States; ^2^ Rehabilitation Institute of Chicago Department of Physical Medicine and Rehabilitation Northwestern University Chicago, IL United States; ^3^ Department of Electrical and Computer Engineering Michigan State University East Lansing, MI United States

**Keywords:** depression, mobile health (mHealth), activities of daily living, cluster analysis, classification

## Abstract

**Background:**

Depression is a common, burdensome, often recurring mental health disorder that frequently goes undetected and untreated. Mobile phones are ubiquitous and have an increasingly large complement of sensors that can potentially be useful in monitoring behavioral patterns that might be indicative of depressive symptoms.

**Objective:**

The objective of this study was to explore the detection of daily-life behavioral markers using mobile phone global positioning systems (GPS) and usage sensors, and their use in identifying depressive symptom severity.

**Methods:**

A total of 40 adult participants were recruited from the general community to carry a mobile phone with a sensor data acquisition app (Purple Robot) for 2 weeks. Of these participants, 28 had sufficient sensor data received to conduct analysis. At the beginning of the 2-week period, participants completed a self-reported depression survey (PHQ-9). Behavioral features were developed and extracted from GPS location and phone usage data.

**Results:**

A number of features from GPS data were related to depressive symptom severity, including circadian movement (regularity in 24-hour rhythm; *r*=-.63, *P*=.005), normalized entropy (mobility between favorite locations; *r*=-.58, *P*=.012), and location variance (GPS mobility independent of location; *r*=-.58, *P*=.012). Phone usage features, usage duration, and usage frequency were also correlated (*r*=.54, *P*=.011, and *r*=.52, *P*=.015, respectively). Using the normalized entropy feature and a classifier that distinguished participants with depressive symptoms (PHQ-9 score ≥5) from those without (PHQ-9 score <5), we achieved an accuracy of 86.5%. Furthermore, a regression model that used the same feature to estimate the participants’ PHQ-9 scores obtained an average error of 23.5%.

**Conclusions:**

Features extracted from mobile phone sensor data, including GPS and phone usage, provided behavioral markers that were strongly related to depressive symptom severity. While these findings must be replicated in a larger study among participants with confirmed clinical symptoms, they suggest that phone sensors offer numerous clinical opportunities, including continuous monitoring of at-risk populations with little patient burden and interventions that can provide just-in-time outreach.

##  Introduction

Depression is a common mental disorder. Estimates of the 12-month prevalence rate for major depressive disorder range from 6.6-10.3%, while lifetime risk has been estimated at 16.6-17.1% [1-3]. Subthreshold depressive symptoms are far more common [4], causing significant impairment in people’s lives and putting them at risk for future mental health concerns [5]. Depression, at both diagnosable and subthreshold levels, imposes a very high societal burden in terms of cost, lost productivity, morbidity, suffering, and mortality [6-8] and is a leading cause of disability and disease burden worldwide [9]. By the year 2020, the World Health Organization estimates that depression will be the second largest cause of “lost years of healthy life” worldwide [10].

Depression is treatable using a variety of methods, including antidepressants and psychotherapy; however, very few people who need treatment receive it [11]. It often takes months or years for depression to be identified and treated in our health care system, when it is treated at all [12]. One of the most common settings where depression is managed is primary care [13,14]; however, primary care physicians might fail to identify most patients with depressive symptoms [15,16]. Thus, more efficient methods of monitoring could significantly improve the delivery of services to those in need.

Mobile phones are becoming the most ubiquitous consumer device in our world. Equipped with powerful sensing, computation, and communication capabilities, mobile phones—specifically smartphones—can continuously monitor an individual’s context including physical activity, location, and environment. Depression is associated with several behavioral components (eg, reduction in activity, psychomotor retardation, changes in sleep) and motivational states (eg, anhedonia), some of which may be detectable using mobile phone sensors [17,18]. Thus, mobile phones hold significant promise as a platform to monitor behavioral and environmental indicators of risk and resilience and to improve long-term management and treatment delivery to people suffering from depression.

Indeed, some work has shown promise in this area. A first study in our group found that phone sensor data could detect social patterns among depressed patients, but this was a small study with only 8 participants [19]. Other groups have found that phone sensors were effective at detecting social and sleep behaviors among patients with depression [20,21], and such features correlated significantly with severity of depressive symptoms [22].

The aim of this study was to extend previous work by focusing specifically on behavioral markers related to movement through geographic space, which we hypothesized would be related to depressive symptom severity, given depression results in decreased motivation, withdrawal, and activity. In addition, excessive use of mobile phones is considered compulsive behavior and has been linked to some symptoms of depression [23,24]. Thus, we also explored the relationship of depression symptom severity to the use of the phone that was used to collect the sensor data. To achieve these aims, we used our mobile phone app, Purple Robot [25,26], to collect global positioning system (GPS) location and phone usage data from participants with varying levels of depression severity. We defined a number of behavioral features based on these data and built classification and regression models to examine their relationship to depression symptom severity.

## Methods

### Participants

We recruited 40 adult participants from April-July 2013 using craigslist advertisements. Participants were eligible if they had an email account, computer, and broadband access to the Internet, were within a cellular network range the majority of the day, were able to speak and read English, were at least 19 years of age, and lived within the United States of America. Participants signed an online consent form, and research staff reviewed the consent over the phone. The study was approved by the Northwestern University Institutional Review Board.

At the beginning of the study, participants were asked to complete an online assessment consisting of a demographics questionnaire and the Patient Health Questionnaire-9 (PHQ-9), a commonly used measure of self-reported depressive symptom severity [27], which produces scores ranging from 0-27. Scores of less than 5 indicate no depression, 5-9 mild depression, 10-14 moderate depression, 15-19 moderately severe, and over 20 severe depression [28].

If participants owned and used an Android device with operating system 2.3 (Gingerbread) or higher, research staff assisted with the download, installation, and configuration of Purple Robot (see the Purple Robot section below). Participants who did not own a compatible phone were given an Android Nexus 4 with Purple Robot installed and configured. Phones were either picked up from the study’s office or mailed directly to the participant.

Participants were instructed to keep the phone with them and charged throughout the day for 2 weeks. In addition, research staff explained that Purple Robot would be collecting GPS location and phone usage data. As part of the purpose of this study was to test Purple Robot’s functionality in the field, research staff checked in periodically, via phone and email, with the participants to ensure the app was working properly and to answer questions.

### Purple Robot

Purple Robot is an open-source Android app that we developed to collect mobile phone sensor data [25,26]. The app implements a store-and-forward architecture wherein the sensor data are gathered, stored on the device, and transmitted as network connectivity becomes available. This allows us to collect data in a variety of wireless connectivity scenarios with the confidence that intermittent network access did not affect the nature, quality, or quantity of the collected data.

Purple Robot anonymizes personally identifiable and other sensitive information before storage and transmission, using standard MD5 hashing and advanced encryption standard [29] algorithms. Once the data are anonymized, they are stored and later transmitted to the data collection server before being deleted from the device. Sensor data residing on the server can be linked with other information gathered during the study only if the unique identifiers used by the participants and the study-specific keys used to encrypt the data are known.

The Purple Robot mobile app and supporting server infrastructure is capable of collecting information about the user’s physical context (eg, motion), social settings (eg, number of Facebook friends), and phone usage behavior (eg, screen state). It also enables us to craft a complete data collection strategy configured for analyzing the relationship between depression and behavior data features of daily life.

In this study, we configured Purple Robot to collect the GPS location and phone usage data, as the aim of this study was to focus on behavioral markers related to movement through space and the phone usage behavior. In our next study, we plan to use Purple Robot to collect data from a variety of phone sensors.

Purple Robot sampled the GPS location sensor once every 5 minutes and collected phone usage data by detecting the screen on and off events.

### Data Preprocessing

The goal of the data preprocessing stage was to facilitate the extraction of features from both the GPS location and the phone usage data.

**Figure 1:**
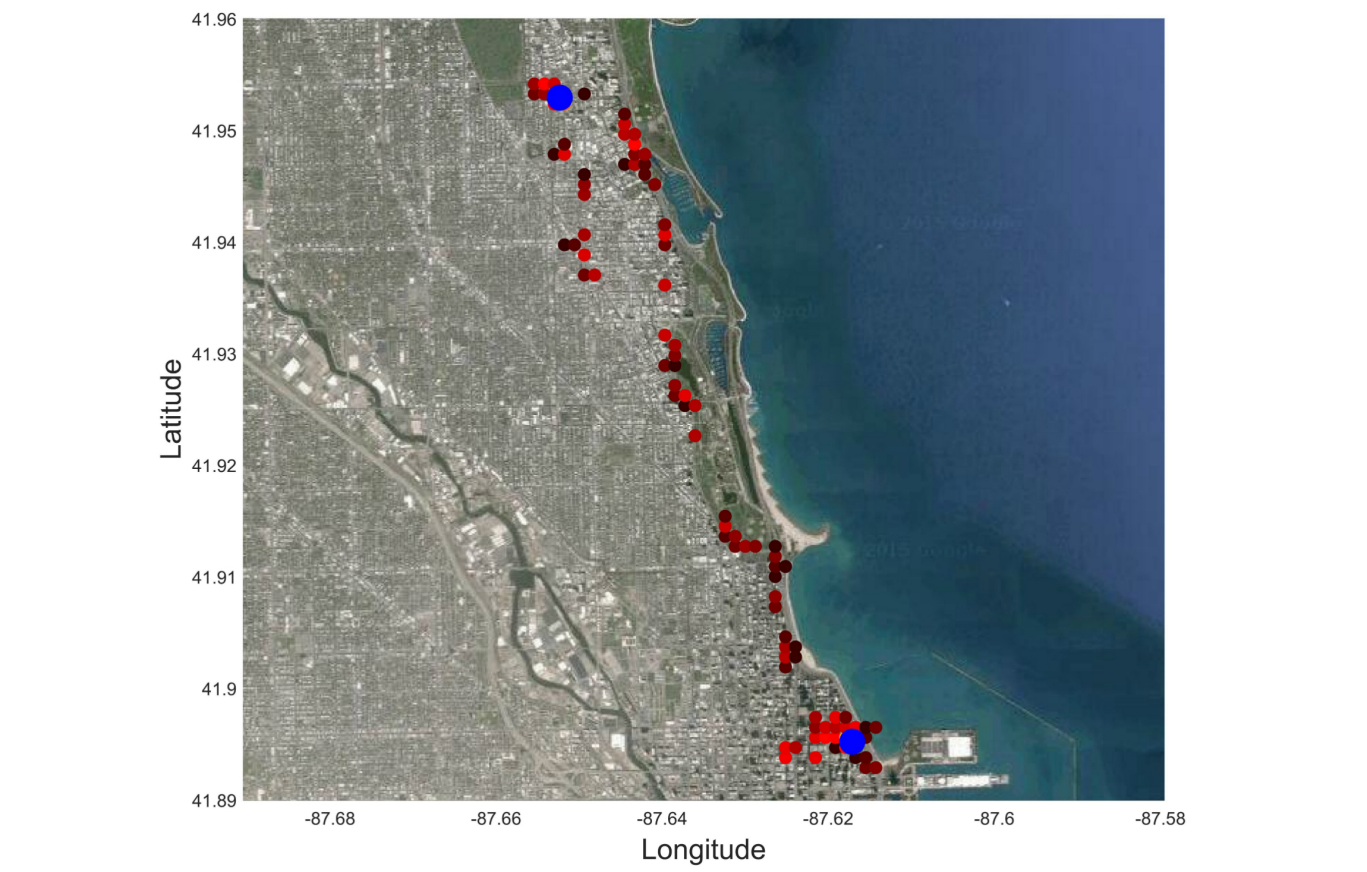
Example GPS location data, overlaid on satellite image. Each small circle represents a histogram bin, which has a size of 500 by 500 meters. The colors indicate the number of samples captured by each bin (brighter means more samples). The bigger blue circles show the center of the clusters detected by the clustering algorithm.

For the location data (Figure 1), we used two procedures. The first procedure determined whether each GPS location data sample came from a stationary state (eg, working in an office) or a transition state (eg, walking on the street). To do so, we estimated the movement speed at each location data sample by calculating its time derivative and then used a threshold speed that defined the boundary between these two states. In this study, we set this threshold to 1 km/h.

The second procedure was clustering. We applied clustering only to the data samples in the stationary state. The goal was to identify the places where participants spent most of their time, such as home, workplaces, parks, etc. We used a distance-based clustering algorithm called *K*-means [30], in which the data were partitioned into *K* clusters such that the overall distances of the data points to the centers of their clusters were minimized. Because the number of clusters was unknown, we started with one cluster and increased the number of clusters until the distance of the farthest point in each cluster to its cluster center fell below a threshold. This threshold determined the maximum radius of a cluster, which was set to 500 meters in our study.

**Figure 2:**
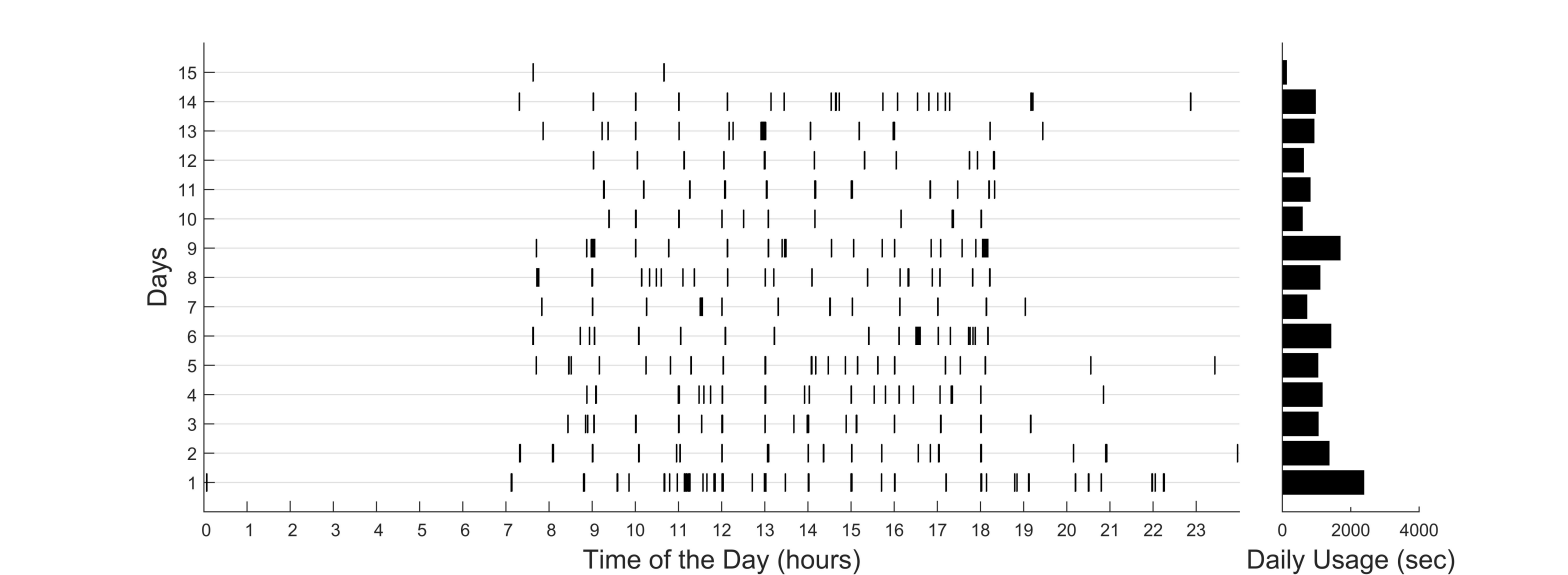
Example phone usage data from a participant. Each row is a day, and the black bars show the extent of time during which the phone has been is use. The bars on the right side show the overall phone usage duration for each day.

Phone usage data were gathered by looking at the periods of time when the phone screen was on (Figure 2). Given that the phone screen would go on when receiving notifications from apps such as text messages, we eliminated brief screen-on events not initiated by the participant that had durations of less than 30 seconds.

### Feature Extraction

#### Location Variance

We defined location variance to measure the variability in a participant’s GPS location. To calculate location variance, we used only the location data of stationary states (see Data Preprocessing). Specifically, location variance was computed as the logarithm of the sum of the statistical variances of the latitude and the longitude components of the location data:

*Location Variance* = log(*σ*_lat_^2^+ *σ*_long_^2^) (1)

We applied the logarithm to compensate for the skewness in the distribution of location variance across participants.

#### Number of Clusters

Number of clusters represented the number of location clusters found by the *K*-means algorithm in the preprocessing stage.

#### Entropy

We defined entropy to measure the variability of the time the participant spent at the location clusters. This feature was developed based on the concept of entropy from information theory [31]. It was calculated as:

*Entropy* = −∑_*i*_ *p*_*i*_log *p*_*i*_(2)

where each *i*=1, 2, …, *N* represented a location cluster, *N* denoted the total number of location clusters, and *p*_*i*_ was the percentage of time the participant spent at the location cluster *i*. High entropy indicated that the participant spent time more uniformly across different location clusters, while lower entropy indicated greater inequality in the time spent across the clusters. For example, if a participant spent 80% of time at home and 20% at work, the resulting entropy would be −(0.8log0.8 + 0.2log0.2) ≈ 0.500, while if they spent 50% at home and 50% at work, the resulting entropy would be −(0.5log0.5 + 0.5log0.5) ≈ 0.693.

#### Normalized Entropy

We defined normalized entropy by dividing the entropy by its maximum value, which is the logarithm of the total number of clusters:

*Normalized Entropy* = *Entropy* ∕ log *N* (3)

Unlike entropy, normalized entropy is invariant to the number of clusters and thus depends solely on the distribution of the visited location clusters. The value of normalized entropy ranges from 0-1, where 0 indicates that all location data points belong to the same cluster, and 1 implies that they are uniformly distributed across all the clusters.

#### Home Stay

Home stay measured the percentage of time a participant spent at home relative to other location clusters. To obtain this measure, we first needed to know which cluster represented the participant’s home. We identified the home cluster based on two heuristics: (1) the home cluster is among the first to the third most visited clusters, and (2) the home cluster is the cluster most visited during the time period between 12 a.m. and 6 a.m. In our dataset, which did not contain participants having night shift work, these two heuristics led to one and only one location cluster for every participant.

#### Circadian Movement

We defined circadian movement to capture the temporal information of the location data. This feature measured to what extent the participants’ sequence of locations followed a 24-hour, or circadian, rhythm. For example, if a participant left home for work and returned home from work around the same time each day, the circadian movement was high. On the contrary, a participant with a more irregular pattern of moving between locations had a lower circadian movement.

To calculate circadian movement, we first used the least-squares spectral analysis, also known as the Lomb-Scargle method [32], to obtain the spectrum of the GPS location data. Then, we calculated the amount of energy that fell into the frequency bins within a 24±0.5 hour period, in the following way:

*E* = ∑_*i*_ psd(*f*_*i*_) ∕ (*i*_*1*_−*i*_*2*_) (4)

where *i* = *i*_1_, *i*_1_+1, *i*_1_+2, …, *i*_2_, and *i*_1_ and *i*_2_ represent the frequency bins corresponding to 24.5 and 23.5 hour periods. psd(*f*_*i*_) denotes the power spectral density at each frequency bin *f*_*i*_. We calculated *E* separately for longitude and latitude and obtained the total circadian movement as:

*CM* = log(*E*_lat_ + *E*_long_) (5)

We applied the logarithm to account for the skewness in the distribution.

#### Transition Time

Transition time represented the percentage of time during which a participant was in a non-stationary state (see Data Preprocessing). This was calculated by dividing the number of GPS location samples in transition states by the total number of samples.

#### Total Distance

Total distance measured the total distance in kilometers taken by a participant. It was calculated by accumulating the distances between the location samples.

#### Phone Usage Frequency

Phone usage frequency indicated, on average, how many times during a day a participant interacted with their phone.

#### Phone Usage Duration

Phone usage duration measured, on average, the total time in seconds that a participant spent each day interacting with their phone.

### Relationship Between Features and Levels of Depression

We performed a preliminary statistical analysis to find out how each feature would correspond to levels of depressive symptoms and whether it would be able to distinguish people with any level of depression from those with none. The former was investigated by correlating each feature with the PHQ-9 score that was obtained at the beginning of the study. The latter was explored first by dividing participants into those with depressive symptoms (PHQ-9 ≥5) and the ones without (PHQ-9 <5). The cutoff of 5 on the PHQ-9 score was used because scores in this range are indicative of “no symptoms” of depression and those who reach this range after treatment are considered to be in full remission.

### Estimating Depression States from Features

#### Score Estimation Model

We used a linear regression model to estimate each participant’s PHQ-9 score using the features extracted from their phone sensor data. The model was defined as the following:

*Depression Score=a_0_+a_i_F_i_+a_2_F_2_+…+a_n_F_n_* (6)

where *n* is the number of features. The coefficients *a*_0_ *, a*_1,_ *… a*_*n*_ were obtained by minimizing the squared error between the estimated and the true PHQ-9 scores (see Model Optimization).

#### Classification Model

We used a logistic regression classifier to identify participants who had symptoms of depression (PHQ-9 ≥5) versus the ones with no symptoms (PHQ-9 <5). This classifier consisted of a linear model and a logistic sigmoid function, *g(x)* = 1 ∕ (1+exp(−*x*)), that generated values between 0 and 1 indicating the probability of the participant having depressive symptoms:

P(*Depressive Symptoms*) = *g*(*b*_0_ + *b*_1_ *F*_1_ + *b*_2_ *F*_2_ + … + *b*_*n*_ *F*_*n*_) (7)

When P(*Depressive Symptoms*) was higher than 0.5, we considered the participant to have depressive symptoms. Otherwise, we considered there was no sign of depression.

We used an optimization procedure to adjust the parameters *b*_0_, *b*_1_, … , *b*_*n*_ (see Model Optimization).

#### Model Optimization

We used the least squares approach to adjust the parameters of both score estimation and classification models. This method performs well as long as the number of features relative to the number of samples is low. Otherwise, the model overfits the data. To minimize the overfitting problem, we used the elastic-net regularization method when the number of features was high.

The elastic-net regularization prevents the coefficient from becoming too large by adding the following penalizing term to the cost function:

*H(K,λ_1_,λ_2_)=λ_1_||K||_1_+λ_1_||K||_2_* (8)

where *K*=*k*_0_, *k*_1_, … , *k*_*n*_ is the vector containing the regression or the classification model parameters, and ‖*K*‖_1_ = ∑_*i*_|*k*_*i*_| and ‖*K*‖_2_ = √∑_*i*_ *k*_*i*_^2^ are its first (L1) and second (L2) norms (*i*=1,2,…,*n*). The coefficients *λ*_1_ and *λ*_2_ are optimized by cross-validating on the training data. Elastic-net regularization has been shown to outperform other regularization methods especially in cases where some of the features are strongly correlated [33].

#### Model Evaluation

To evaluate regression and classification models, we created 1000 bootstrapped sets of features and their corresponding PHQ-9 scores. Then we trained and cross-validated the models on each set using the leave-one-participant-out method.

To assess the performance of each score estimation model, we calculated the normalized root mean square deviation (NRMSD), which measures the percent difference between the PHQ-9 scores estimated by the model on the test participants and their true scores. We used the observed range of PHQ-9 scores, which was 0-17, to normalize the NRMSDs. To evaluate the performance of each classification model, we evaluated its accuracy, sensitivity, and specificity in identifying participants with depressive symptoms as compared to the ground truth.

## Results

### Participant Characteristics and Adherence

All 40 participants completed the 2 weeks of the study. However, due to insufficient sensor data for a number of participants, we considered 28 of them for the data analysis. Among these, 18 were considered for GPS location and 21 for phone usage data analysis. These two analyses were performed independently.

The 12 excluded participants did not provide sufficient GPS location and/or phone usage data for our analysis, meaning that their data were not available for more than 50% of the time. The reasons for unavailability of data included problems in the connection between the early version of Purple Robot and the data server, patients not charging their phones, and unavailability of any network connections for a long period of time for certain locations.

The 28 participants included in the analysis were 20 females and 8 males. Their ages ranged from 19-58, with a mean of 28.9 (SD 10.1). Their PHQ-9 scores ranged from 0-17, with a mean of 5.57 (SD 4.9). Of the 28 participants, 14 (50%) had no signs of depression (PHQ-9 <5) with an average PHQ-9 score of 1.5 (SD 1.34), and the other 14 participants (50%) were in the mild to severe range (PHQ-9 ≥5), with an average PHQ-9 score of 9.64 (SD=3.54). Participants who were not included in any analyses due to inadequate data were not statistically different from these 28 participants in their age, gender, or PHQ-9 scores.

Among the participants who were considered for location data analysis, 9 had depressive symptoms and 9 did not. For the ones considered for phone usage data analysis, these numbers were 10 and 11, respectively.

### Relationship Between Features and Levels of Depression

GPS location and phone usage sensor features were calculated as described in Feature Extraction. The number of location clusters that was found by the *K*-means algorithm ranged from 1-9, with an average of 4.06. The average daily phone usage duration across the participants was about 41 minutes (SD 57 minutes) with an average daily usage frequency of 14.2 times (SD 8.69).

**Figure 3:**
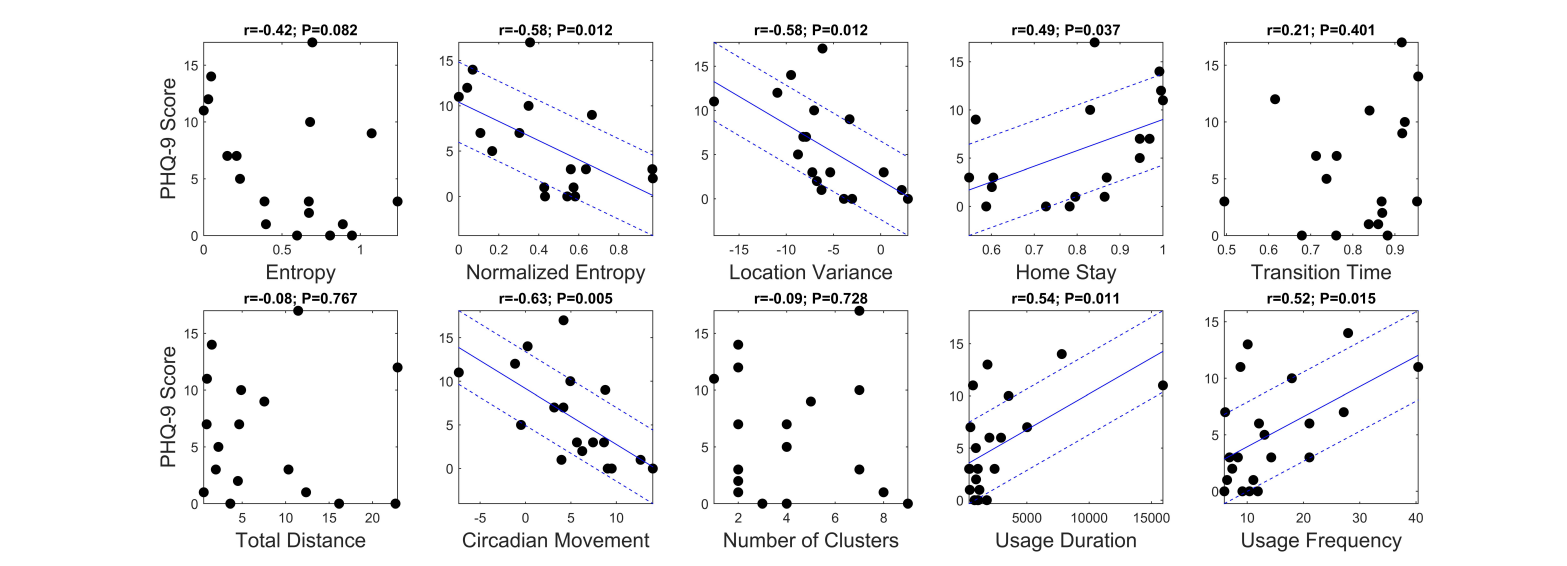
Scatter plots for location and phone usage data versus PHQ-9 scores, respectively. The coefficient of correlation between each feature and PHQ-9 scores and its corresponding P-value is shown on top of each plot. Solid and dashed lines, shown only for strong correlations (*P*<.05), show the fitted regression model and +/- root mean square deviation from the model, respectively.

The correlation analysis between the features and the PHQ-9 scores revealed that 6 of the 10 features were significantly correlated to the scores (Figure 3). Specifically, circadian movement, normalized entropy, and location variance showed strong correlations with Pearson’s correlation coefficients of -.63, -.58, and -.58, respectively. Both phone usage features, usage duration and usage frequency, were also significantly correlated with *r*=-.54 and 0.52, respectively.

**Figure 4:**
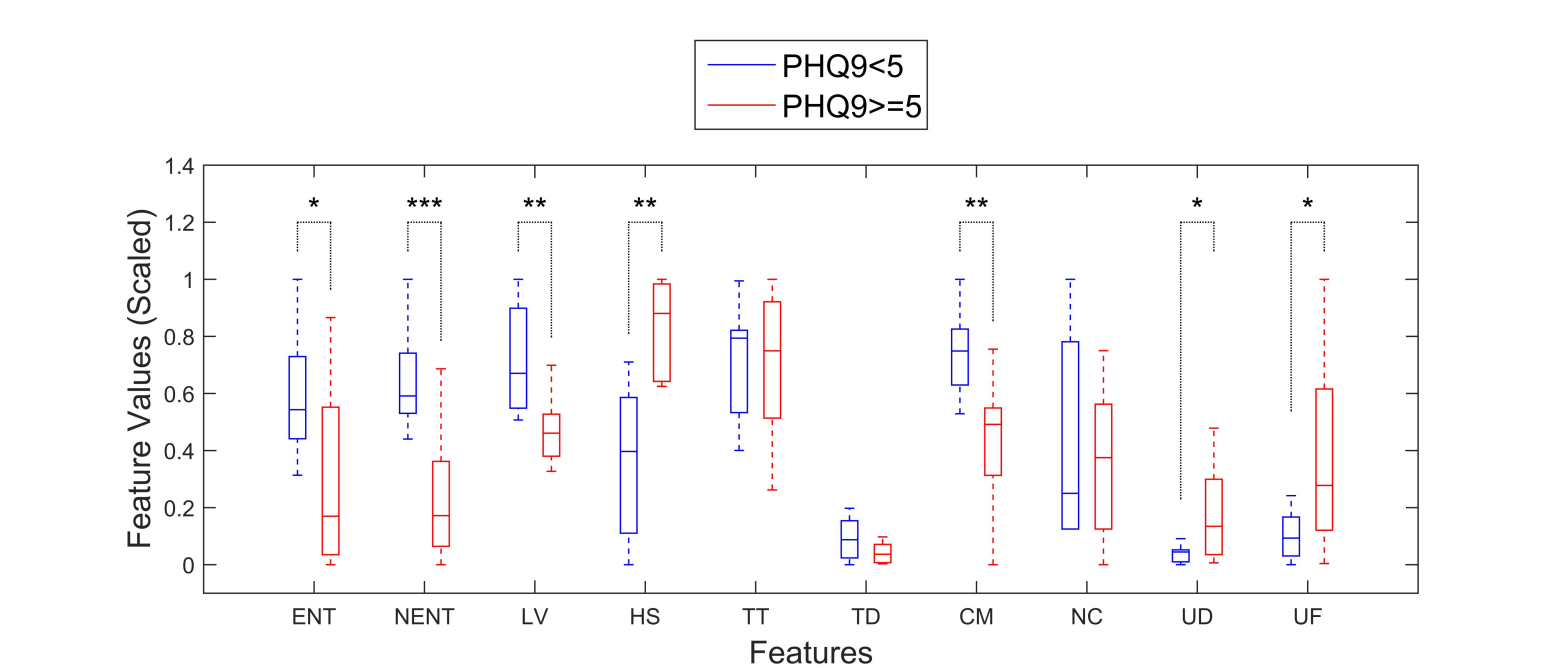
Comparison of location and usage feature statistics between participants with no symptoms of depression (blue) and the ones with (red). Feature values are scaled between 0 and 1 for easier comparison. Boxes extend between 25th and 75th percentiles, and whiskers show the range. Horizontal solid lines inside the boxes are medians. One, two, and three asterisks show significant differences at *P*<.05, *P*<.01, and *P*<.001 levels, respectively (ENT, entropy; ENTN, normalized entropy; LV, location variance; HS, home stay; TT, transition time; TD, total distance; CM, circadian movement; NC, number of clusters; UF, usage frequency; UD, usage duration).

The *t* tests between participants with depressive symptoms and the ones without (Figure 4) also revealed that the value of the same six features (circadian movement, normalized entropy, location variance, home stay, phone usage duration, and phone usage frequency) were significantly different between the participants with no sign of depression (PHQ-9 <5) and the rest (PHQ-9 ≥5).

**Figure 5:**
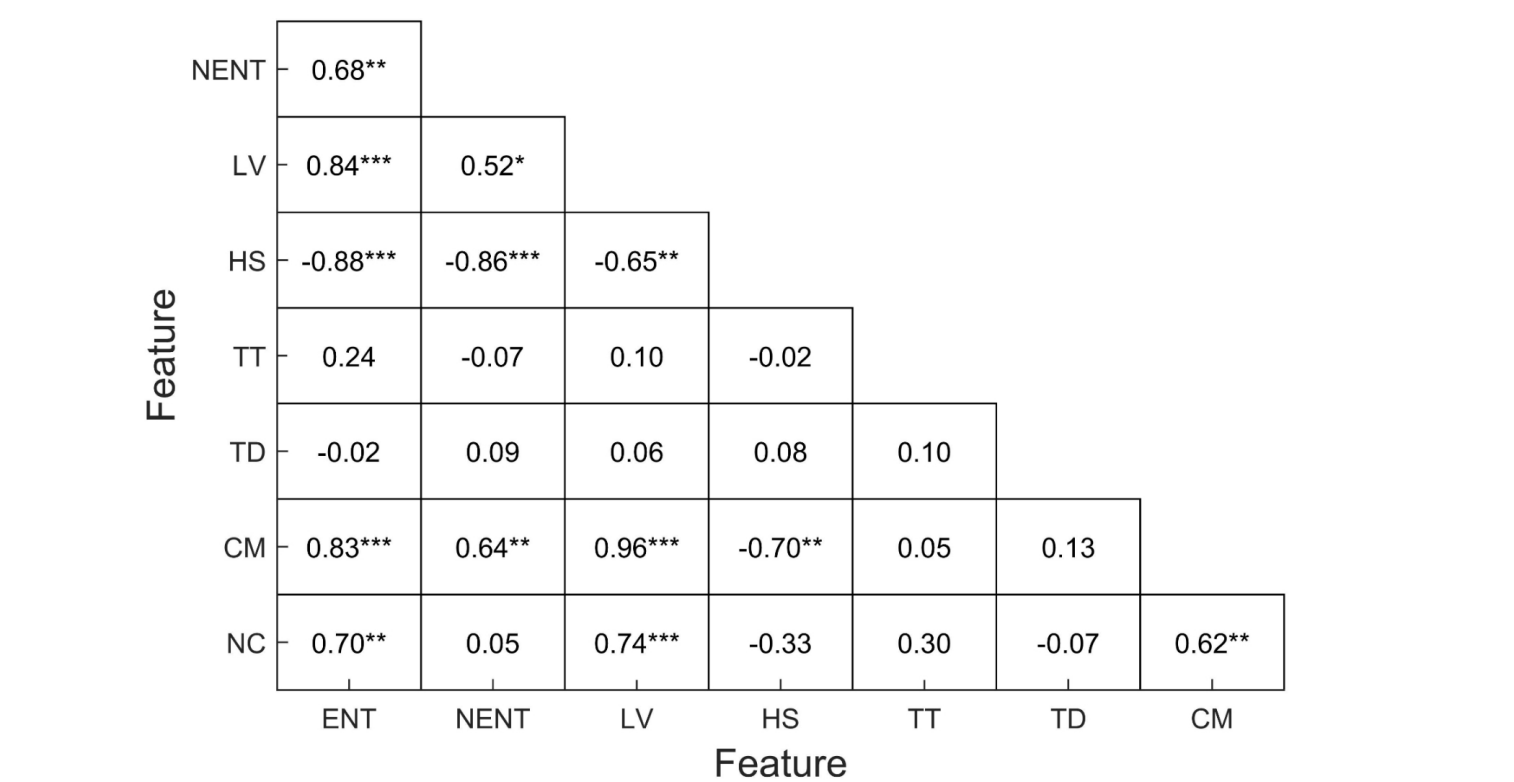
Coefficients of correlation between location features. One, two, and three asterisks indicate significant correlation levels at P<.05, P<.01, and P<.001, respectively (ENT, entropy; ENTN, normalized entropy; LV, location variance; HS, home stay; TT, transition time; TD, total distance; CM, circadian movement; NC, number of clusters).

A correlation analysis across the features revealed that a number of them were highly correlated (Figure 5). Noticeably, there was a significant correlation between normalized entropy, location variance, and home stay. This is not surprising, as all these variables measure the amount of movement through space in different ways. However, the significant correlation between circadian movement and location variance is interesting and indicates that participants with more mobility also had more regular patterns of movement. The correlation between phone usage duration and frequency was also high (*r*=.89).

### Estimating Depression States from Location Features

The results of the earlier statistical analysis suggested that we may be able to estimate an unseen subject’s depression state using some of our features. To test this hypothesis, we trained and cross-validated score prediction and classification models (Equations 6-7) using the procedure described in Model Evaluation.

We trained the models first on each individual feature and then all features combined together. As the results (Table 1) show, the models trained on the features that had stronger correlations with PHQ-9 scores were better able to distinguish the participants with depressive symptoms from those who had none. Columns 2-4 show the cross-validated accuracy, sensitivity, and specificity of each classification model (Equation 7) in classifying participants into the ones with and without depressive symptoms. Column 5 shows the cross-validated NRMSDs of the PHQ-9 score estimation models (Equation 6). Specifically, the four features normalized entropy, location variance, home stay, and circadian movement achieved the lowest NRMSDs and highest accuracies. These performances, however, did not improve by combining the features. This can be the result of some unavoidable overfitting as the number of input variables increases, which leads to a poor generalization.

**Table 1:** Classification of participants with and without depressive symptoms and estimating their PHQ-9 scores using location features individually and aggregated.

	Classification (PHQ9<5 vs PHQ9≥5)			PHQ-9 score estimation
Training features	% mean accuracy (SD)	% mean sensitivity	% mean specificity	Mean NRMSD (SD)
Entropy	69.7 (3.5)	66.8	72.7	0.262 (0.017)
Normalized entropy	86.5 (3.4)	88.4	84.9	0.235 (0.016)
Location variance	75.7 (4.6)	80.2	71.5	0.229 (0.014)
Home stay	75.9 (4.9)	80.5	71.7	0.253 (0.015)
Transition time	41.1 (9.2)	43.4	38.7	0.303 (0.020)
Total distance	56.4 (6.6)	69.6	43.4	0.343 (0.041)
Circadian movement	78.6 (4.1)	80.1	77.5	0.222 (0.014)
Number of clusters	41.5 (8.9)	47.4	35.5	0.305 (0.022)
All	78.8 (6.2)	83.6	74.5	0.251 (0.023)

### Estimating Depression States from Phone Usage Features

We performed the same analyses on the phone usage features. Since the number of these features (n=2) was much smaller than the number of samples (n=21), both score prediction and classification models could be directly applied to the combined feature space without using elastic-net regularization. The results (Table 2) show that each of the usage frequency and usage duration features could provide acceptable accuracies and NRMSDs without further improvement by aggregating them. Columns 2-4 show the cross-validated accuracy, sensitivity, and specificity of each classification model (Equation 7) in classifying participants into the ones with and without depressive symptoms. Column 5 shows the cross-validated NRMSDs of the PHQ-9 score estimation models (Equation 6).

**Table 2:** Classification of participants with and without depressive symptoms and estimating their PHQ-9 scores using phone usage features individually and aggregated.

	Classification (PHQ9<5 vs PHQ9≥5)			PHQ9 score estimation
Training features	% mean accuracy (SD)	% mean sensitivity	% mean specificity	Mean NRMSD (SD)
Usage duration	74.2 (3.4)	64.0	83.9	0.268 (0.018)
Usage frequency	68.6 (4.1)	56.4	79.6	0.249 (0.013)
All	65.7 (4.9)	55.7	74.9	0.273 (0.019)

## Discussion

### Principal Findings

This study reported on the potential to use commonly available mobile phone sensor data, including GPS and phone usage, to identify depressive symptom severity. We extracted a number of semantically meaningful features from these data and found a strong correlation between a number of them and the PHQ-9 scores. These features included normalized entropy, location variance, home stay, circadian movement, and phone usage duration and frequency. By training score estimation models on each of these six features, we could estimate the PHQ-9 scores of unseen participants with a relatively low error (NRMSD). In addition, classifiers trained on these features were able to discriminate between those with and those without symptoms with a high degree of accuracy, good sensitivity, and specificity.

The normalized entropy feature measured the frequency with which a person visited different locations and the distribution of that frequency across locations. The high negative correlation that was found between this feature and the PHQ-9 scores indicated that people with greater depressive symptom severity visited fewer locations and were more likely to favor some locations over others. Part of this was likely due to the increased amount of time people with depressive symptoms spent at home, measured by the home stay feature. The finding for the location variance feature, on the other hand, indicated that people with depressive symptoms tend to move less through geographic space. These findings are consistent with the patterns of loss of motivation, decreased activity, and social withdrawal that characterize depression [17,18].

The finding for the circadian rhythm of movement through space fits into well-established literature investigating alterations to the wake-sleep patterns associated with depression [34,35]. These data suggest that disruptions in behavioral patterns during waking hours include not only the volume of activity but may also extend to the patterns of behavior. These pattern changes may be due to the genetic and hormonal factors [36] that have been implicated in depression-related circadian rhythm changes or may be a result of low motivation and decreased organization.

Phone usage data were also strongly correlated to depressive symptom severity. Greater levels of depressive symptom severity were related to greater phone usage duration and frequency. This observation is supported by a number of previous studies that have found a correlation between mobile phone use and some depressive symptoms [23,24]. However, we should note that phone usage in this context was defined as any interaction with the phone, and we were not able to isolate the specific types of interactions (eg, using apps, texting). Thus, it is difficult to determine which specific behaviors were related to symptom severity.

### Limitations

While we believe that our study has revealed some of the daily-life correlates of depression that can be captured by mobile phones, the results are very preliminary, and a number of caveats must be mentioned. First, this study examined only the association between self-reported depressive symptoms and features derived from location and phone usage data. Thus, we cannot infer any causal relationship here. In fact, while the PHQ-9 is a well-validated measure of depression, we cannot exclude the possibility that factors other than depressive symptoms are responsible for these relationships. For example, the results may be due to other unmeasured factors, such as chronic illness or dispositional factors, which result both in depressive symptoms and differences in behavioral patterns.

Second, while some participants demonstrated levels of depressive symptoms consistent with clinical levels of depression, this was a small sample that was not necessarily representative of typical trends seen in people with depression. Future research could recruit more representative participants with depression and match them on characteristics that might impact one’s pattern of movements through geographic locations (eg, occupational status, size of social network, or chronic health problems).

Finally, we did not attempt to correct for the possible effect of multiple comparison. However, given our interest in exploring potential indicators of depressive symptoms, the increased likelihood of Type II errors introduced by such corrections might undermine important features. A major goal of such corrections is to increase confidence in one’s findings and given the preliminary nature of our results, we urge future efforts to cross-validate these relationships in larger-scale investigations. Nevertheless, we believe the computation of behaviorally meaningful features (eg, normalized entropy, circadian movement) and the relationship of these features with depression found in this study might provide a valuable starting place for subsequent investigations of the use of sensor data for the monitoring and the detection of depression.

### Conclusions

Regardless of these limitations, the ability to passively detect behavioral factors related to depression, such as activity levels and their patterns, opens the possibility of a new generation of behavioral intervention technologies that can passively detect and positively reinforce behaviors that are likely to improve depression (eg, engagement in activities that provide pleasure, a sense of accomplishment, or involve social engagement) and offer support when risk states are detected (eg, withdrawal, staying at home). This can improve the identification of depression and the ability of health care settings to allocate resources to those in need and overcome the individual and systemic barriers to conventional psychological treatment [37].

The use of phone sensors allows the capture of information that is potentially indicative of depressive symptoms without the use of questionnaires or requiring the person to use special devices. Phones fit into the fabric of people’s lives. People tend to keep phones with them all or most of the time, and phones can provide data unobtrusively and with no effort on the part of the user. This capacity offers new opportunities to identify human behavior patterns associated with depression or other health and mental health disorders. Furthermore, behavioral features might be more sensitive to changes in a person’s daily life that indicate early benefit from treatment or highlight potential areas for improvement. As such, as these features and the link between them and depression become better understood, they may play an important role in understanding the progression of depression and its response to treatment.

## Abbreviations

GPS: global positioning system
NRMSD: normalized root mean square deviation
PHQ-9: Patient Health Questionnaire-9
